# The differences of clinical characteristics and outcomes between imported and local patients of COVID-19 in Hunan: a two-center retrospective study

**DOI:** 10.1186/s12931-020-01551-5

**Published:** 2020-11-26

**Authors:** Chang Wang, Lizhi Zhou, Juan Chen, Yong Yang, Tianlong Huang, Min Fu, Ya Li, Daniel M. George, Xiangyu Chen

**Affiliations:** 1 Department of Nephrology, The Second Xiangya Hospital, Central South University, Changsha, 410011 Hunan China; 2 Department of Urology, The Second Xiangya Hospital, Central South University, Changsha, 410011 Hunan China; 3 Department of Radiology, The Central Hospital of Xiangtan, Xiangtan, 410011 Hunan China; 4 Department of Intensive Care Unit, The Central Hospital of Changsha, Nanhua University, Hengyang, 410011 Hunan China; 5 Department of Orthopaedics, The Second Xiangya Hospital, Central South University, Changsha, 410011 Hunan China; 6 Department of Neurology, the Fourth Hospital of Changsha, Changsha, 410011 Hunan China; 7 Department of Radiology, The Second Xiangya Hospital, Central South University, Changsha, 410011 Hunan China; 8 Royal Adelaide Hospital Adelaide, Adelaide, SA Australia

**Keywords:** COVID-19, SARS-CoV-2, Clinical characteristics, Outcomes, Virulence

## Abstract

**Background:**

The clinical characteristics and outcomes of the 2019 novel coronavirus (COVID-19) pneumonia are different in Hubei compared to other regions in China. But there are few comparative studies on the differences between imported and local patients which may provide information of the different courses of the virus after transmission.

**Methods:**

We investigated 169 cases of COVID-19 pneumonia in two centers in Hunan Province, and divided them into two groups according to epidemiological history, "imported patients" refers to patient with a clear history of travel in Wuhan within 14 days before onset, and " local patients” refers to local resident without a recent history of travel in Wuhan, aiming to analyze the difference in clinical characteristics and outcomes between the two groups. All the epidemiological, clinical, imaging, and laboratory data were analyzed and contrasted.

**Results:**

The incidence of fever on admission in imported patients was significantly higher than local patients. There was a significantly higher proportion of abnormal pulmonary signs, hypokalemia, hyponatremia, prolonged PT, elevated D-dimer and elevated blood glucose in imported patients. Compared with local patients, the proportion using antibiotics, glucocorticoids and gamma globulin were significantly higher in imported patients. The moderate type was more common in local patients, and the severe type were more frequent in imported patients. In addition, the median duration of viral clearance was longer in imported patients.

**Conclusions:**

In summary, we found that imported cases were more likely to develop into severe cases, compared with local patients and required more powerful treatments.

*Trial registration* Registered 21st March 2020, and this study has been approved by the Medical Ethics Committee (Approved Number. 2020017).

## Background

The outbreak of COVID-19 pneumonia was recognized in December 2019 and the World Health Organization declared 2019-nCoV epidemic as a Public Health Emergency of International Concern on January 30th, 2020 [1]. As of June 15th, 2020, China has confirmed 84,778 patients and 4645 deaths [2]. Among them, 1019 patients were confirmed, and four patients died in Hunan [3]. The pathogen that caused the outbreak now is officially named SARS-CoV-2, which is a member of the coronavirus family [4].

Compared with the studies in Wuhan and other provinces and cities in China, we found that Wuhan has a higher rate of severe patients, higher mortality, and longer hospitalization time [5–7]. Previous studies suggest that most viruses will co-evolve with their hosts, and intermediate virulence maximizes pathogen fitness, which is a trade-off between virulence and transmission [8]. Excluding the influence of environmental factors, has the virulence of the SARS-CoV-2 been reduced in the process of transmission? Regretfully, few studies focus on this area at present, which is conducive to our comprehensive understanding of the clinical and epidemiological characteristics of COVID-19.

Hunan is geographically adjacent to Hubei, and many people have escaped into Hunan before Wuhan closed the city borders [9], which may lead to many potentially infected patients. Since it is relatively difficult to distinguish the generations of patients clinically, we adopted two classifications: “imported patients” refers to patients with a clear history of travel in Wuhan within 14-days before onset, while “local patients” refers to local residents without a recent history of travel in Wuhan.

In this study, we collected data from 169 adult COVID-19 pneumonia patients from two centers in Hunan Province. All patients were divided into imported patients and local patients according to epidemiology history. We made a comprehensive comparison and analysis of their clinical features and outcomes to explore the differences between the two groups.

### Methods

#### Study design and participants

This is a two-center, retrospective study. We enrolled 169 adult patients of COVID-19 pneumonia admitted to hospital in Changsha and Xiangtan, Hunan Province from January 21st, 2020 to February 21st, 2020. Examining the physiological differences between children and adults, however, the differences in clinical characteristics between children and adult cases of COVID-19 pneumonia patients has been confirmed [10], we therefore abandoned the inclusion of underage patients. The enrolled patients included 134 patients from Changsha Public Health Treatment Center and 35 patients from Xiangtan Central Hospital. Both hospitals are the only designated hospitals to treat COVID-19 pneumonia patients in Changsha and Xiangtan respectively. All patients were diagnosed as COVID-19 pneumonia according to the guidelines issued by the National Health Commission of the People’s Republic of China (PRC) [11] and divided into imported patients (74 patients) and local patients (95 patients) according to their epidemiological history. All the clinical data was collected separately up to March 2nd, 2020.

### Procedures

All patients were diagnosed according to the guidelines of the National Health Commission of the PRC [11], all suspected patients were tested with nasal/pharyngeal swabs by reverse transcription polymerase chain reaction (RT-PCR) immediately. If the nucleic acid test was positive, patients would be registered as confirmed.

We collected the basic information (gender, age, epidemiological history, underlying diseases, etc.), clinical characteristics and signs (fever, respiratory symptoms, gastrointestinal symptoms, respiratory frequency, fatigue, etc.), laboratory data (blood biochemistry, coagulation function, blood gas analysis, liver and kidney function assessment, infection-related biomarkers, etc.), imaging results (ground-glass opacity, consolidation, involved scope, pleural effusion, etc.), and treatment measures (respiratory support, antiviral drugs, antibiotics, hormones, etc.), from the hospital medical records. The data were as of March 2nd, 2020. If important data was missing or clarification was needed, we obtained the data by communicating with the attending clinicians. For imaging data, we used two commercial multi-detector CT scanners (LightSpeed, GE Medical Systems, USA; SOMATOM, Siemens Medical, Germany) to perform chest CT scans in all patients. CT images were collected during one or two breath holds. Imaging features include the type and distribution of lesions, the number of lesions and the lobes involved. All CT images were read by two radiologists with more than 10 years' experience, and if differences arose, a third radiologist was added for analysis.

Before the data was collected, the attending clinicians had classified all patients into four categories: mild, moderate, severe and critical types, according to the guidelines of the National Health Commission of the PRC [11]. We followed the attending clinicians' classification.

### Outcomes

The primary outcome was death. The secondary outcomes included the incidence of serious complications, utilization rate of mechanical ventilation and the duration to negative RT-PCR tests.

### Definitions

Fever is defined as the temperature equal or higher than 37.3℃, slight fever is 37.3–38.0 ℃, and high fever is > 39.0 ℃. Anemia is defined as hemoglobin lower than 120 g/L. Hypoalbuminemia is the level of albumin lower than 40.0 g/L. The level of serum sodium lower than 135 mmol/L is defined as hyponatremia.

Oxygen therapy: Including nasal cannula oxygen therapy, high-flow nasal cannula oxygen therapy, non-invasive positive pressure ventilation and invasive positive pressure ventilation. All the patients were administered oxygen therapy, except the patients did not accept. The indication for setting up extracorporeal membrane oxygenation (ECMO): patient with invasive positive pressure ventilation, (1) with a FiO_2_ > 90%, the oxygenation index is less than 80 mmHg, lasts for more than 3–4 h; (2) airway platform pressure ≥ 35 cmH_2_O [11].

Confirmed standard [11]:

- Nasal/Pharyngeal swabs or other samples were positive for viral nucleic acid tested by RT-PCR,
- The gene sequencing of the virus was highly homologous to the known novel coronavirus 2019.
  Clinical classification standard [11]:
- Mild: Clinical symptoms are slight, with no pneumonia manifestations seen on imaging,
- Moderate type: With symptoms such as fever and difficulty breathing, with pneumonia manifestations seen on imaging,
- Severe: Shortness of breath, RR > 30 beats/min, or oxygen saturation < 93% at rest, or arterial blood oxygen partial pressure (Pa0_2_)/oxygen concentration (Fi0_2_) < 300 mmHg, or lung imaging demonstrating lesion progression more than 50% within 24–48 h,
- Critical: Respiratory failure requiring mechanical ventilation, or shock, or multiple organ failure requiring ICU monitoring and treatment.

### Statistical analysis

For continuous variables, we expressed them as a mean with standard deviation (SD) or median with interquartile range (IQR). We assessed the differences by two-sample T-test or Mann–Whitney test. Categorical variables were expressed as counts (%), and differences were tested by χ^2^ or Fisher exact tests, if appropriate. For the laboratory results, we also evaluated whether the values were beyond the normal range. We used SPSS (version 26.0) for all analyses, *p* < 0.1 was considered statistically significant, due to the small sample size.

## Results

### Demographics, baseline and clinical characteristics of COVID-19 pneumonia patients (Table 1)

**Table 1:** Demographics, baseline and clinical characteristics of COVID-19 pneumonia patients

Variables	All cases (n = 169)	Imported cases (n = 74)	Local cases (n = 95)	p value
Age (year)				
Median (IQR)	45 (34.5–55)	45 (35–53.5)	43 (34–57)	0.872
Range				
19–49	106 (62.7)	50 (67.6)	56 (58.9)	0.392
50–64	38 (22.5)	13 (17.6)	25 (26.3)	
≥ 65	25 (14.8)	11 (14.9)	14 (14.7)	
Sex				
Male	86 (50.9)	40 (54.1)	46 (48.4)	0.536
Female	83 (49.1)	34 (45.9)	49 (51.6)	
Temperature on admission (°C)				
Median (IQR)	36.8 (36.5–37.4)	37.0 (36.6–37.5)	36.8 (36.5–37.2)	0.076
Range				
< 37.3	120 (71.0)	46 (62.1)	74 (77.9)	0.065
37.3–38.0	37 (21.9)	21 (28.4)	16 (16.8)	
38.1–39.0	11 (6.5)	7 (9.5)	4 (4.2)	
> 39	1 (0.6)	0 (0)	1 (1.1)	
Pulmonary auscultation on admission				
Crackles	9 (5.3)	5 (6.8)	4 (4.2)	0.507
Stridor	0 (0)	0(0)	0 (0)	1.000
Both	3 (1.8)	3 (4.1)	0 (0)	0.082
Complication				
Not less than one	53 (31.4)	19 (25.7)	34 (35.8)	0.183
Recurrent respiratory infections	4 (2.4)	1 (1.4)	3 (3.2)	0.632
Hypertension	19 (11.2)	6 (8.1)	13 (13.7)	0.329
Diabetes	13 (7.7)	4 (5.4)	9 (9.5)	0.393
Immunodeficiency	2 (1.2)	0 (0)	2 (2.1)	0.505
Chronic obstructive pulmonary disease	3 (1.8)	1 (1.4)	2 (2.1)	1.000
Cancer	2 (1.2)	1 (1.4)	1 (1.1)	1.000
Cardiovascular and cerebrovascular diseases	10 (5.9)	2 (2.7)	8 (8.4)	0.189
Symptoms or signs				
Fever	123 (72.8)	58 (78.4)	65 (68.4)	0.167
Cough	136 (80.5)	64 (86.5)	72 (75.8)	0.117
Expectoration	71 (42.0)	28 (37.8)	43 (45.3)	0.350
Hemoptysis	4 (2.4)	3 (4.1)	1 (1.1)	0.320
Nasal congestion	3 (1.8)	1 (1.4)	2 (2.1)	1.000
Headache	23 (13.6)	7 (9.5)	16 (16.8)	0.183
Shortness of breath	37 (21.9)	20 (27.0)	17 (17.9)	0.190
Muscle soreness	11 (6.5)	3 (4.1)	8 (8.4)	0.351
Fatigue	73 (43.2)	32 (43.2)	41 (43.2)	1.000
Gastrointestinal symptoms	53 (31.4)	22 (29.7)	31 (32.6)	0.740
Onset-visit interval (days)				
Median (IQR)	3 (1–6)	3 (1–6)	3 (1–6)	0.711

Data are n (%) or median (IQR), unless otherwise specified

In our study, 74 (43.7%) patients had a clear history of living in Wuhan before the onset of the disease, including Wuhan locals and those working, studying, or traveling in Wuhan. These patients who were infected with the virus in Wuhan were regarded as imported patients, while the rest were regarded as local Hunan patients. There were no medical staff in our patient population.

The median age of all patients was 45-years (IQR 34.5 to 55). The median age of imported patients was 45-years, and local patients was 43-years. The age of the patients was mainly between 19 and 49-years (67.6% of imported patients and 58.9% of local patients). The proportion of local patients over 50-years old (41%) was slightly higher than that of imported patients (32.5%). There was no significant difference in gender between the two groups. The male-to-female ratio of all patients was close to 1:1, which was consistent with the study of Wu et al. [12].

On admission, the body temperature for most patients was below 37.3 ℃. Among the fevers, most of them were considered slight fevers, and only 1 case had a high fever. The incidence of fever in imported patients (37.9%) was significantly higher than that in local patients (22.1%, *p* = 0.065).

The abnormal physical signs on pulmonary auscultation were largely crackles or mixed crackles and stridor. The proportion of abnormal pulmonary signs was significantly higher in imported patients (10.9% vs 4.2%). Only three of the imported patients had mixed crackles and stridor compared with none of the local patients.

Among all the patients, 31.4% had at least one comorbidity. Compared with local patients, imported patients had a higher proportion of underling comorbidities (35.8% vs 25.7%), no statistical significance was found. The top three comorbidities were hypertension (8.1% vs 13.7%), diabetes (5.4% vs 9.5%), and cardiovascular and cerebrovascular diseases (2.7% vs 8.4%) in each group.

In this study, cough (86.5% vs 75.8%), fever (78.4% vs 68.4%) and fatigue (both 43.2%) were still the three most common clinical symptoms of COVID-19 pneumonia in both groups. However, the proportion of gastrointestinal symptoms (such as nausea, vomiting, diarrhea, etc.) was also close to one third (29.7% vs 32.6%), which is not as low as the previous study [7]. However, no statistical significance was observed between the two groups. In addition, the median symptom onset-visit interval of both imported and local patients was 3-days, with no significant difference.

### Radiographic findings of COVID-19 patients (Table 2)

**Table 2:** Radiographic findings of COVID-19 patients

Variables	All cases (n = 169)	Imported cases (n = 74)	Local cases (n = 95)	p value
CT findings on admission^a^				
Ground-glass opacity (GGO) only	130/166 (78.3)	57/73 (78.1)	73/93 (78.5)	1.000
Consolidation only	1/166 (0.6)	0/73 (0)	1/93 (1.1)	1.000
Mixed GGO and consolidation	21/166 (12.7)	10/73 (13.7)	11/93 (11.8)	0.815
Involved scope^a^				
Unilateral pneumonia	19/166 (11.4)	6/73 (8.2)	13/93 (14.0)	0.328
Bilateral pneumonia	136/166 (81.9)	62/73 (84.9)	74/93 (79.6)	0.421
CT findings after treatment^a^				
Ground-glass opacity (GGO) only	81/163 (49.7)	39/72 (54.2)	42/91 (46.2)	0.346
Consolidation only	1/163 (0.6)	0/72 (0)	1/91 (1.1)	1.000
Mixed GGO and consolidation	53/163 (32.5)	21/72 (29.2)	32/91 (35.2)	0.501

Data are n (%), n/N (%) or median (IQR), unless otherwise specified

^a^Data were missing for the item in several cases

On admission, radiographs demonstrated that 130 of the 166 patients (78.3% vs 78.1%) had ground-glass opacity (GGO), and 21 of the 166 patients (13.7% vs 11.8%) had mixed GGO and consolidation. One patient had consolidation only. The proportion of bilateral pneumonia was higher in imported patients (84.9% vs 79.6%) while the proportion of unilateral pneumonia was higher in local patients (8.2% vs 14.0%). However, no significant difference was observed.

When treated for 5–7 days after admission, the proportion of patients with only GGO decreased (54.2% vs 46.2%), while the proportion of mixed GGO and consolidation patients increased (29.2% vs 35.2%) in both groups, which conforms to the natural variation of lung imaging performance.

### Laboratory results of COVID-19 patients (Table 3)

**Table 3:** Laboratory results of COVID-19 patients

Variables	Normal range	All cases (n = 169)	Imported cases (n = 74)	local cases (n = 95)	p value
Blood routine					
Leucocytes^a^ (× 10^9^/L)	3.5–9.5	4.62 (3.63–5.89)	4.61 (3.44–5.99)	4.62 (3.64–5.87)	0.844
Increased		4/168 (2.4)	1 (1.4)	3/94 (3.2)	0.631
Decreased		38/168 (22.6)	19 (25.7)	19/94 (20.2)	0.459
Neutrophils^a^ (× 10^9^/L)	1.8–6.3	2.89 (2.30–3.67)	2.80 (2.26–3.56)	2.93 (2.30–3.77)	0.693
Increased		9/168 (5.4)	4 (5.4)	5/94 (5.3)	1.000
Decreased		17/168 (10.1)	6 (8.1)	11/94 (11.7)	0.608
Lymphocytes^a^ (× 10^9^/L)	1.1–3.2	1.13 (0.81–1.55)	1.11 (0.81–1.53)	1.18 (0.81–1.60)	0.666
Increased		2/168 (1.2)	0 (0)	2/94 (2.1)	0.504
Decreased		80/168 (47.6)	36 (48.6)	44/94 (46.8)	0.877
Hemoglobin^a^ (g/L)	120–160	130.5 (120.0–141.8)	132.0 (121.0–143.0)	129.0 (117.8–141.0)	0.150
Decreased		41/168 (24.4)	15 (20.3)	26/94 (27.7)	0.284
Platelets^a^ (× 10^9^/L)	125–350	175.5 (145.0–227.0)	172.5 (139.0–228.0)	181.5 (145.8–227.3)	0.699
Increased		3/168 (1.8)	1 (1.4)	2/94 (2.1)	1.000
Decreased		24/168 (14.3)	12 (16.2)	12/94 (12.8)	0.658
Coagulation function					
Prothrombin time^a^ (s)	9.4–12.5	11.8 (11.0–12.7)	12.1 (11.2–12.7)	11.7 (10.8–12.5)	0.099
Increased		44/167 (26.3)	22 (29.7)	22/93 (23.7)	0.384
Activated partial thromboplastin time^a^ (s)	25.1–36.5	33.1 (30.4–36.1)	32.9 (31.0–35.6)	33.3 (30.2–36.8)	0.943
Increased		40/167 (24.0)	15 (20.3)	25/93 (26.9)	0.364
Decreased		5/167 (3.0)	0 (0)	5/93 (5.4)	0.067
D-dimer^a^ (mg/L)	0.0–0.5	0.31 (0.15–0.57)	0.36 (0.19–0.59)	0.26 (0.13–0.53)	0.072
Increased		50/167 (29.9)	26 (35.1)	24/93 (25.8)	0.234
Blood gas analysis					
pH^a^	7.35–7.45	7.47 (7.45–7.50)	7.47 (7.44–7.51)	7.48 (7.45–7.50)	0.785
Increased		109/159 (68.6)	45/70 (64.3)	64/89 (71.9)	0.309
Decreased		1/159 (0.6)	0/70 (0)	1/89 (1.1)	1.000
PaO_2_^a^ (mmHg)	75–105	87.4 (71.6–109.0)	90.7 (70.8–109.4)	86.0 (72.0–108.4)	0.429
Decreased		45/159 (28.3)	19/70 (27.1)	26/89 (29.2)	0.860
PaCO_2_^a^ (mmHg)	35–46	37.0 (32.9–40.6)	36.6 (33.6–39.7)	37.5 (32.8–41.3)	0.718
Increased		8/159 (5.0)	4/70 (5.7)	4/89 (4.5)	0.732
Decreased		59/159 (37.1)	26/70 (37.1)	33/89 (37.1)	1.000
Blood biochemistry					
Albumin (g/L)	40.0–55.0	38.8 (35.6–42.7)	37.87 (35.43–42.36)	39.64 (36.30–43.30)	0.133
Decreased		95 (56.2)	46 (62.2)	49 (51.6)	0.211
Alanine aminotransferase (U/L)	9–50	19.9 (13.9–29.3)	20.9 (13.6–30.5)	19.7 (14.0–28.3)	0.611
Increased		18 (10.7)	10 (13.5)	8 (8.4)	0.322
Aspartate aminotransferase (U/L)	15–40	22.5 (17.3–28.6)	23.2 (16.0–29.1)	21.9 (17.5–28.2)	0.837
Increased		20 (11.8)	10 (13.5)	10 (10.5)	0.634
Creatine kinase (U/L)	< 171	68.5 (40.9–103.9)	72.0 (39.9–108.6)	65.0 (41.0–90.5)	0.712
Increased		17 (10.1)	9 (12.2)	8 (8.4)	0.450
Creatine kinase-MB (U/L)	< 25	9.8 (6.3–12.3)	10.3 (6.1–13.1)	9.7 (6.4–12.9)	0.947
Increased		10 (5.9)	4 (5.4)	6 (6.3)	1.000
Total bilirubin (μmol/L)	3.4–17.1	10.7 (7.8–15.3)	11.5 (7.8–17.8)	10.1 (7.6–14.3)	0.128
Increased		38 (22.5)	20 (27.0)	18 (18.9)	0.266
Serum potassium (mmol/L)	3.5–5.5	4.10 (3.69–4.44)	3.95 (3.63–4.40)	4.15 (3.85–4.48)	0.058
Increased		2 (1.2)	1 (1.4)	1 (1.1)	1.000
Decreased		18 (10.7)	10 (13.5)	8 (8.4)	0.322
Serum sodium (mmol/L)	135–145	137.5 (135.6–139.8)	137.0 (134.8–139.8)	137.8 (136.1–139.9)	0.088
Increased		1 (0.6)	1 (1.4)	0 (0)	0.438
Decreased		30 (17.8)	19 (25.7)	11 (11.6)	0.025
Blood urea nitrogen (mmol/L)	2.8–7.5	4.26 (3.42–5.30)	4.38 (3.36–5.33)	4.13 (3.45–5.30)	0.560
Increased		14 (8.3)	6 (8.1)	8 (8.4)	1.000
Serum creatinine (μmol/L)	57.0–111.0	53.68 (41.48–68.00)	51.94 (40.30–66.23)	55.00 (44.98–72.90)	0.156
Increased		5 (3.0)	1 (1.4)	4 (4.2)	0.387
Lactate dehydrogenase (U/L)	120–250	169.0 (140.1–216.1)	176.6 (137.7–226.8)	160.1 (141.4–207.5)	0.264
Increased		23 (13.6)	13 (17.6)	10 (10.5)	0.258
Glucose^a^ (mmol/L)	3.9–6.1	5.63 (4.95–7.44)	6.10 (5.07–7.75)	5.41 (4.86–6.95)	0.049
Increased		69/168 (41.1)	37 (50.0)	32/94 (34.0)	0.041
Infection-related biomarkers					
C-reactive protein (mg/L)	< 10	12.23 (3.25–26.06)	12.71 (4.25–28.52)	10.30 (2.30–25.37)	0.225
Increased		91 (53.8)	43 (58.1)	48 (50.5)	0.354
Procalcitonin^a^ (ng/mL)	< 0.5	—^b^	—^b^	—^b^	—^b^
Increased		3/168 (1.8)	2/73 (2.7)	1/95 (1.1)	0.581

Data are n (%), n/N (%) or median (IQR), unless otherwise specified

^a^Data were missing for the item in several cases

^b^Not all data were collected by quantitative data

Leukopenia accounted for 22.6% (25.7% vs 20.2%), lymphocytopenia 47.6% (48.6% vs 46.8%), anemia 24.4% (20.3% vs 27.7%), thrombocytopenia 14.3% (16.2% vs 12.8%), but there was no significant difference between the two groups at admission.

The Prothrombin Time (PT) in imported patients (median 12.1 s) was longer than local patients (median 11.7 s). Also, the proportion of increased D-dimer in imported patients was significantly higher (0.35 mg/L vs 0.26 mg/L), *p* < 0.1.

The results of blood gas analysis in the two groups were similar. Alkalosis was present in 68.6% of the patients, while 28.3% of the patients had a decrease in PaO_2_ and 37.1% had a decrease in PaCO_2_. No significant difference was found.

Most patients had hypoalbuminemia (56.2%), of which the albumin level of imported patients (median 37.87 g/L, 62.2%) is lower than that of local patients (median 39.64 g/L, 51.6%), but there are no significant difference. The prevalence of abnormal serum ALT was 11.8% and AST was 10.7%, while that of CK and CK-MB was less than 10%, but there was still no significant difference.

There was a significant difference in hyponatremia 25.7% vs 11.6% (*p* = 0.025). The median serum potassium and median serum sodium of imported patients (3.95 mmol/L and 137.0 mmol/L respectively) were significantly lower than those of local patients (4.15 mmol/L and 137.8 mmol/L), while the proportion of hyperglycemia was significantly higher than that of local patients (50% vs 34%, *p* < 0.1).

In addition, C-reactive protein (CRP) was elevated in more than half of the patients (58.1% vs 50.5%). The median CRP of imported patients was 12.71 mg/L, while the median CRP of local patients was 9.92 mg/L, but there was no statistical difference. Surprisingly, only three patients had increased procalcitonin.

### Complications, treatments and clinical outcomes of COVID-19 patients (Table 4)

**Table 4:** Complications, treatments and clinical outcomes of COVID-19 patients

Variables	All cases (n = 169)	Imported cases (n = 74)	Local cases (n = 95)	*p* value
Clinical classification				
Mild type	10 (5.9)	5 (6.8)	5 (5.3)	0.750
Moderate type	130 (76.9)	52 (70.3)	78 (82.1)	0.097
Severe type	24 (14.2)	15 (20.3)	9 (9.5)	0.074
Critical type	5 (3.0)	2 (2.7)	3 (3.2)	1.000
Severe complication				
ARDS	16 (9.5)	9 (12.2)	7 (7.4)	0.304
Shock	2 (1.2)	2 (2.7)	0 (0)	0.190
AKI	4 (2.4)	1 (1.4)	3 (3.2)	0.632
MODS	3 (1.8)	1 (1.4)	2 (2.1)	1.000
Treatments				
Admitted in ICU	20 (11.8)	10 (13.5)	10 (10.5)	0.634
Oxygen therapy	162 (95.9)	71 (95.9)	91 (95.8)	1.000
High-flow nasal cannula oxygen therapy	14 (8.3)	7 (9.5)	7 (7.4)	0.780
Prone Positioning	4 (2.4)	2 (2.7)	2 (2.1)	1.000
Non-invasive positive pressure ventilation	7 (4.1)	3 (4.1)	4 (4.2)	1.000
Invasive positive pressure ventilation	3 (1.8)	2 (2.7)	1 (1.1)	0.582
Extracorporeal membrane oxygenation	2 (1.2)	2 (2.7)	0 (0)	0.190
Continuous renal replacement therapy	1 (0.6)	1 (1.4)	0 (0)	0.438
Antiviral treatment				
Monotherapy	41 (24.3)	16 (21.6)	25 (26.3)	0.588
Combination therapy	128 (75.7)	58 (78.4)	70 (73.7)	0.588
Antibiotic treatment	87 (51.5)	45 (60.8)	43 (44.2)	0.043
Glucocorticoids	44 (26.0)	27 (36.5)	17 (17.9)	0.008
Course of treatment (days) -Median (IQR)	7 (5–9)	7 (5–10)	6 (4–8.5)	0.259
Gamma globulin	44 (26.0)	27 (36.5)	17 (17.9)	0.008
Course of treatment (days)-Median (IQR)	6 (4–8)	6 (4–7)	6 (3.5–8.5)	0.913
Clinical outcome				
Discharged from hospital	154 (91.1)	72 (97.3)	82 (86.3)	0.002
Death	1 (0.6)	1 (1.4)	0 (0)	
Hospitalization	14 (8.3)	1 (1.4)	13 (13.7)	
Length of hospital stay (days)	13 (11.0–18.0)	13 (11.0–17.8)	13 (10.0–19.0)	0.916
Viral clearance duration (days) ^a^				
Median (IQR)	10 (8–15)	11 (8–15)	9 (7–15)	0.080

Data are n (%), or median (IQR), unless otherwise specified

^a^ Patients without symptoms before onset were exclude

The moderate type of COVID-19 was the most common (70.3% vs 82.1%), followed by severe, mild, and the critical type. The proportion of severe type of imported patients (20.3%) was significantly higher than that of local patients (9.3%), while the proportion of moderate type was significantly lower (70.3% vs 81.4%), *p* < 0.1.

During hospitalization, 18 patients had severe complications, such as acute respiratory distress syndrome (ARDS), shock, acute kidney injury (AKI), and multiple organ dysfunction syndrome (MODS). The incidence of ARDS in imported patients (12.2%) was higher than that in local patients (7.4%). Shock only occurred in imported patients (2.7%). Twenty patients were admitted to the ICU, of which 13.5% were imported patients and 10.5% were local patients. Oxygen therapy was administered in about 95% of patients in both groups. There were two patients treated with ECMO and one patient was treated with continuous renal replacement therapy, all were imported patients. No statistical difference was observed.

All patients received antiviral therapy during hospitalization, with about 25% receiving monotherapy and 75% receiving combination therapy. The most used antiviral drugs were Lopinavir/Ritonavir, interferon and Arbidol. The patients received monotherapy were using Lopinavir/Ritonavir or Arbidol, and the patients received combination therapy were using Lopinavir/Ritonavir and Arbidol or interferon. The proportion of monotherapy or combination therapy between two groups don’t have significantly difference.

The use of antibiotics, glucocorticoids, and gamma globulins was significantly higher in the imported patients than in local patients (60.8% vs 44.2%, 36.5% vs 17.9%, 36.5% vs 17.9%, respectively), p < 0.05. The antibiotics included Moxifloxacin, Cefoperazone, Piperacillin, and Meropenem.

The type of glucocorticoid was methylprednisolone, with the usual initial dose of 40 mg, and the maximum dose of 80 mg. The dose of gamma globulin was calculated by 0.25 g per kilogram. Glucocorticoids and gamma globulins were not used unless a panel discussion by experts considered them necessary (e.g., ARDS). The median course of treatment of the glucocorticoid was 7-days, while the median course of gamma globulins was 6-days. The median viral clearance duration in imported patients was significantly longer than that in local patients (11 days vs 9 days, *p* = 0.080).

As of March 2nd, 2020, a total of 154 patients were discharged, one died (imported case), and 14 (1 imported case and 13 local patients) were still in hospital. Since Wuhan was closed on 23rd January, there were no more imported patients after the 5th of February, therefore, the patients admitted to the hospital at the later stages were local patients. The median length of hospital stay was 13-days.

## Discussion

This is a retrospective study to explore whether there is a difference between imported cases and local cases of COVID-19. We included 169 patients from Changsha and Xiangtan and divided them into imported patients and local patients according to their epidemiological history. Most patients had a mild fever during the disease. There were higher proportions of fever and abnormal pulmonary auscultation in imported patients.

The clinical classification of mild, moderate, severe, and critical were used. The proportion of the severe type in imported patients was higher, while the proportion of moderate type was lower, indicating that imported patients had more severe clinical manifestations than local patients. In terms of laboratory tests, in imported patients, the proportions of hypokalemia, hyponatremia, prolonged PT, elevated D-dimer and blood glucose were higher, which may be related to the more serious disturbances of the internal environment caused by the infection. In addition, there were higher proportions of hypoproteinemia, lymphocytopenia, elevated CRP, and elevated LDH in imported patients, without significant difference, which are the most common clinical biochemical abnormalities found in COVID-19 pneumonia [7]. These findings demonstrate that imported patients are more likely to progress to the severe type of COVID-19.

Another finding in this study was that more antibiotics were used in imported patients. The most common antibiotic used was moxifloxacin, which is a broad-spectrum antibiotic, suggesting that the imported patients may have a higher rate of superimposed bacterial infections compared to local patients. In addition, the proportions of using glucocorticoids and gamma globulins in imported patients were also higher, suggesting that the infection and incidence of inflammatory storms is more serious in imported patients.

In this study, the viral clearance duration was longer in imported patients. The median viral clearance duration was 11-days in imported patients and 9-days in local patients, which was close to that of Chen et al. [13]. Chen et al. found that the viral clearance duration in patients admitted into ICU was longer than those not admitted into ICU. In other words, the median viral clearance duration may take longer in the more severe cases. We speculate that the negative nucleic acid tests may be related to the viral load of patients, Dr. Liu and his colleagues have identified that the viral load is positively correlated to the clinical manifestations and biochemical indicators in patients [14], which may explain to some extent why imported patients are more serious than local patients.

For the geographical adjacent to Hubei, the total number of infected people in Hunan and Henan were two of the largest in China except Hubei, but the mortality of COVID-19 pneumonia patients in Hunan was 0.39% and in Henan was 1.73%, which were significantly lower than that in Hubei [15]. The insufficient knowledge of the virus and disease, running out of medical resources, only severe patients can be admitted to the hospital at the beginning of the outbreak of COVID-19 pneumonia in Hubei, which may led to the much higher proportion of severe patients and higher mortality. Based on our study, where medical resources were available for all, still found that the clinical manifestations and the ratio requiring special treatment was higher in the imported patients compared with local patients.

A recent study has shown that the incubation period of SARS-CoV-2 in tertiary patients is longer than that in primary and secondary patients, and the viral load is lower, so the infectivity of SARS-CoV-2 in tertiary patients may gradually decrease [16]. This study also mentioned that there were no significant differences in early clinical signs and symptoms between primary, secondary, and tertiary patients, which are consistent with the results of our current study. Another study presented a linear correlation between viral load and severity of lung injury [14]. Therefore, we speculate that the virulence of SARS-CoV-2 may have decreased during the infection of local patients. The clinical characteristics of imported and local patients are not significantly different in the early stages, but as the disease progresses, the imported patients tended to develop more serious symptoms than local patients, indicating that closer observation and earlier intervention for the imported cases may be required.

Psychological problems may be another important factor contributing to the difference between imported and local patients [17]. Lima et al. found that patients are prone to mental health problems during this epidemic, especially in Wuhan [18]. Hence, during the epidemic, all people associated with Wuhan were easily regarded as "black sheep" and suffered discrimination [19]. The imported patients in this study correlate to the local patients in Wuhan to some extent, they were more likely to suffer prejudice from the local people leading to increased psychological problems and detrimental health outcomes.

There are several limitations to our study. First, the sample size is small, which leads to limited statistical difference in some comparisons. Secondly, the mechanism of SARS-CoV-2 virulence decrease still needs further study. Thirdly, we did not have long-term follow-up that can evaluate the long-term prognosis of these patients. Finally, we only included two centers in Hunan Province, so large-scale multicenter studies are needed to verify our findings.

## Conclusions

We found that imported COVID-19 pneumonia patients had a higher tendency to develop into the severe type of pneumonia compared with local patients and required more aggressive treatment. In view of the fact that SARS-CoV-2 has become a global epidemic [2], as there is currently limited effective medicine and treatment, it is important to pay attention to the imported population and quarantine for a while to prevent local spread. Ongoing support for medical resources in epidemic areas is paramount to prevent the collapse of the medical system in these overrun areas.

## Data Availability

The datasets used and/or analysed during the current study are available from the corresponding author on reasonable request.

## Abbreviations

COVID-19: 2019 Novel coronavirus disease
SARS-CoV: Severe acute respiratory syndrome-related coronavirus
SARS-CoV-2: Severe acute respiratory syndrome-related coronavirus-2
CT: Computed tomographic
IQR: Interquartile ranges
GGO: Ground-glass opacity
CK: Creatine kinase
CK-MB: Creatine kinase-MB
AKI: Acute kidney injury
MODS: Multiple organ dysfunction syndrome
ARDS: Acute respiratory distress syndrome
ICU: Intensive care unit
ECMO: Extracorporeal membrane oxygenation
ALT: Alanine aminotransferase
AST: Aspartate aminotransferase
PCT: Procalcitonin
CRP: C-reactive protein
PT: Prothrombin time
LDH: Lactate dehydrogenase
APTT: Activated partial thromboplastin time
